# Supplementary material for: Estimating the Volume of Nodules and Masses on Serial Chest Radiography Using a Deep-Learning-Based Automatic Detection Algorithm: A Preliminary Study
Source: Diagnostics (Basel). 2023 Jun 14;13(12):2060. doi: 10.3390/diagnostics13122060 (PMC10297196; doi:10.3390/diagnostics13122060)
Supplement: Supplementary file 1 [file diagnostics-13-02060-s001.zip › diagnostics-2383482-supplementary.pdf]

Supplementary Material Figure S1: relationship between the volume of the 3D object and the area of the 2D projection

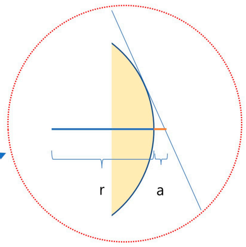

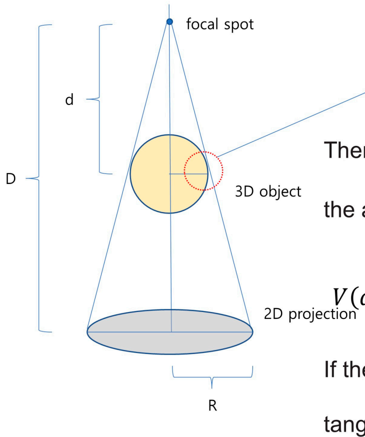

There would be a following relationship between the volume (V) of the 3D object and the area (A) of the 2D projection (α, β, γ, δ are coefficients).

$$V(d, D, a, A) \propto \alpha \ \times A^{\frac{3}{2}} + \beta \times A^{1} + \gamma \times A^{\frac{1}{2}} + \delta$$

If the focal spot moves left and right, it is expressed as a product of sine, cosine or tangent functions, and it will affect the size and shape of the 2D projection, but it is expected that it will not affect the maximum order as a function of area.

Supplementary Material Figure S2: Residual plot for the unit-adjusted multivariable regression model for nodule volume prediction.

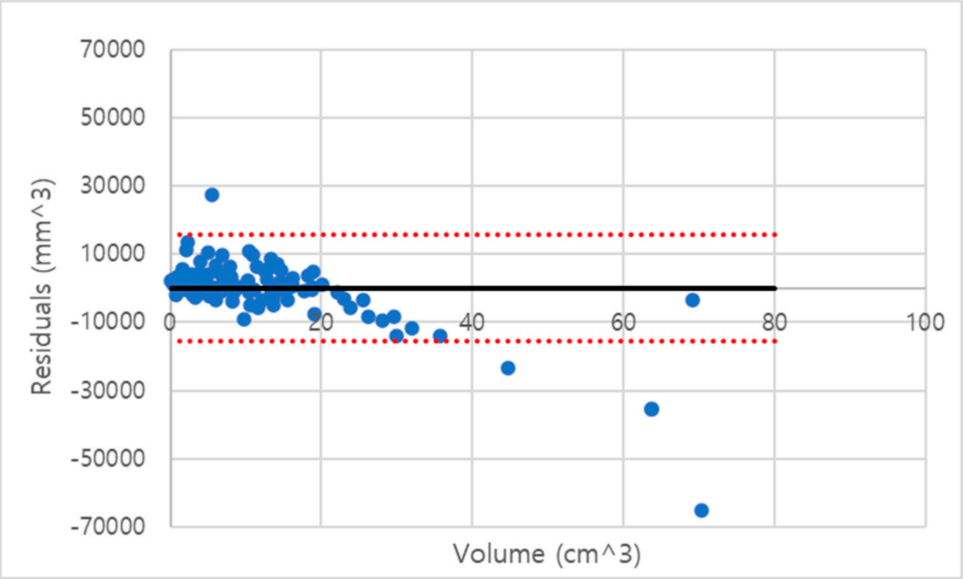

Note: Red dotted lines refer to the lower and upper bounds of 95% confidence intervals for residuals.
